# Cardiac High-Energy Phosphate Metabolism Alters with Age as Studied in 196 Healthy Males with the Help of 31-Phosphorus 2-Dimensional Chemical Shift Imaging

**DOI:** 10.1371/journal.pone.0097368

**Published:** 2014-06-18

**Authors:** Regina Esterhammer, Gert Klug, Christian Wolf, Agnes Mayr, Sebastian Reinstadler, Hans-Josef Feistritzer, Bernhard Metzler, Michael F. H. Schocke

**Affiliations:** 1 Department of Radiology, Medical University Innsbruck, Innsbruck, Austria; 2 Department of Internal Medicine III, Division of Cardiology, Medical University Innsbruck, Innsbruck, Austria; 3 Department of Radiology, District Hospital Reutte, Ehenbichl, Austria; Mayo Clinic, United States of America

## Abstract

Recently published studies have elucidated alterations of mitochondrial oxidative metabolism during ageing. The intention of the present study was to evaluate the impact of ageing on cardiac high-energy phosphate metabolism and cardiac function in healthy humans. 31-phosphorus 2-dimensional chemical shift imaging (31P 2D CSI) and echocardiography were performed in 196 healthy male volunteers divided into groups of 20 to 40 years (I, n = 43), 40 to 60 years (II, n = 123) and >60 years (III, n = 27) of age. Left ventricular PCr/β-ATP ratio, myocardial mass (MM), ejection fraction and E/A ratio were assessed. Mean PCr/β-ATP ratios were significantly different among the three groups of volunteers (I, 2.10±0.37; II, 1.77±0.37; III, 1.45±0.28; all p<0.001). PCr/β-ATP ratios were inversely related to age (r^2^ = −0.25; p<0.001) with a decrease from 2.65 by 0.02 per year of ageing. PCr/β-ATP ratios further correlated with MM (r = −0.371; p<0.001) and E/A ratios (r = 0.213; p<0.02). Moreover, E/A ratios (r = −0.502, p<0.001), MM (r = 0.304, p<0.001), glucose-levels (r = 0.157, p<0.05) and systolic blood pressure (r = 0.224, p<0.005) showed significant correlations with age. The ejection fraction did not significantly differ between the groups. This study shows that cardiac PCr/β-ATP ratios decrease moderately with age indicating an impairment of mitochondrial oxidative metabolism due to age. Furthermore, MM increases, and E/A ratio decreases with age. Both correlate with left-ventricular PCr/β-ATP ratios. The findings of the present study confirm numerous experimental studies showing an impairment of cardiac mitochondrial function with age.

## Background

31-phosphorus magnetic resonance spectroscopy (31P MRS) is a unique tool to investigate human myocardial high-energy phosphate (HEP) metabolism *in vivo*. The ratios between phosphocreatine (PCr) and adenosine-triphosphate (ATP) obtained by 31P MRS are mainly used as an important physiological index for cardiac energy metabolism [1]. The myocardial HEP metabolism is characterized by a remarkable metabolic stability maintaining almost constant levels of PCr and ATP during increases of workload. This metabolic homeostasis, also known as “stability paradox”, is enabled by a complex cellular regulation of mitochondrial respiration, which has extensively reviewed by Saks et al. [2]. Consequently, the PCr/ATP ratio reflects the creatine rephosphorylation rate and, therefore, the mitochondrial function in the myocardium. Mitochondrial insufficiency can be caused by defects in key mitochondrial enzymes, increased mitochondrial proton leak, impaired supply of reducing equivalents or insufficient mitochondrial PO2 [1]. Previous 31P MRS studies have shown that cardiac PCr/ATP ratios are significantly but unspecifically reduced in ischemic and structural heart diseases [3]–[8] as well as in diabetes and other metabolic disorders [9]–[11]. Furthermore, several previous studies have shown that the cardiac PCr/ATP ratios are clearly reduced in patients suffering from hereditary disorders with mitochondrial involvement [12]–[17].

Our study group has shown that cardiac high-energy metabolism correlates positively with exercise capacity and negatively with cardiovascular risk factors [18]–[20]. We also detected a significant effect of age on cardiac high-energy metabolism, showing a decrease in left ventricular PCr/ATP ratio with age [21]. At this time, data on impact of ageing on myocardial PCr/ATP ratios were controversial. Okada et al. as well as Kostler et al. detected reduced PCr and ATP concentrations in elderly but not reduced PCr/ATP ratios, whereby the number of subjects enrolled in each of both studies was relatively small [22]; [23]. The latter study group calculated absolute concentrations for PCr and γ-ATP, demonstrating moderate decreases in both with age, but not in the ratio. A recent study, however, supported our findings and reported also on a decrease in left-ventricular PCr/ATP ratios with age in 49 healthy subjects [24].

At the present time, it is well known that mitochondrial function becomes impaired and declines with age [25]. First, decreased mitochondrial oxidative phosphorylation has been supposed to contribute to impaired cellular metabolism during ageing [26]. Furthermore, myocardial lysosomes also suffer from different alterations with increasing age leading to an impaired autophagocytosis of defective mitochondria due to an overload of heavy lipofuscine and decreased efficiency of lysosomal enzymes [27]; [28]. Both mitochondrial and lysosomal damages are supposed to result in functional heart failure and death of cardiac myocytes. Moreover, it is known that ageing is associated with reduction in early-to-atrial peak ratio [29]; [21] probably caused by accumulation of collagen within the myocardium resulting in increased myocardial stiffness [30]. End-diastolic and end–systolic volumes as well as longitudinal left ventricular function decrease with age, whereas myocardial mass and mass-to-volume index increase [31].

The purpose of this study was to sample left-ventricular PCr/β-ATP ratios derived from the healthy, male subjects serving as control groups in previous publications of our study group. Our hypothesis is that we obtain a clearly significant correlation between age and left-ventricular PCr/β-ATP ratios.

## Results

### Study Population

Summing up two collectives, 196 healthy, asymptomatic male volunteers with a mean age of 47.6±11.2 years (range: 20–70 years) were enrolled into this study. The mean PCr/β-ATP ratio was 1.80±0.40 (range: 0.93–3.22) and the mean EF 61±6%.

**Figure 1:**
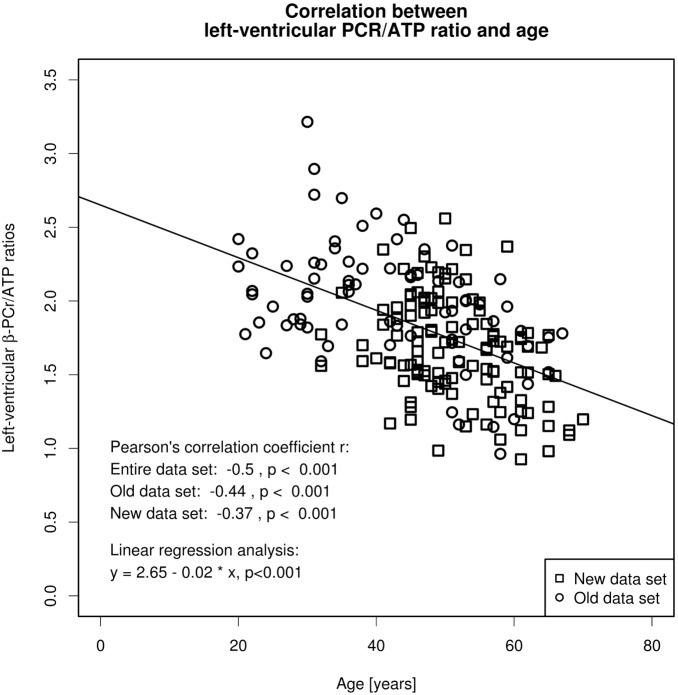
The scatter plot demonstrates the inverse linear relationship between age and left-ventricular phosphocreatine (PCr) to adenosine-triphosphate (ATP) ratio in 196 healthy volunteers. Correlation tests between left-ventricular PCr/β-ATP ratios and age were performed for the entire data set as well as the old (already published in 2003) and the new data set by using the Pearson’s correlation coefficient r. In addition, a linear regression analysis was done for the entire data set.

Since the first collective (median age, 42 years; range 20–67 years; n = 76) contained more younger volunteers than the second collective (median age, 50 years; range, 32–70 years; n = 120), we tested an age-matched selection of both collectives by including all subjects ≥48 years for significant differences in PCr/β-ATP ratios and age. Between these groups, the t-test did not reveal any significant differences in PCr/β-ATP ratios (1.72±0.35 vs. 1.62±0.36, p = 0.237) and age (55±5.2 vs. 55.9±5.9 years, p = 0.948). Furthermore, we performed separately a correlation analysis in both collectives using the Pearson’s correlation coefficient r. As shown in Figure 1, we detected significant correlations between left-ventricular PCr/β-ATP ratios and age in both collectives.

For further evaluations of age-related differences, we divided our volunteers in three groups separated by age: group I, 20–40 years, n = 43 (22%); group II, 40–60 years, n = 126 (64%); group III, >60 years, n = 27 (14%). The metabolic, echocardiographic and clinical data of the three groups are listed in Table 1.

**Table 1:** The clinical and demographical data of the volunteers separated for the different age groups are given.

	20–40 years	40–60 years	>60 years	p values
				I vs II/I vs III/II vs III
Age [years]	30±6	49±5	63±3	**<0.001/<0.001/<0.001**
PCr/ATP	2.10±0.37	1.77±0.36	1.45±0.27	**<0.001/<0.001/<0.001**
EF [%]	63±6	61±5	59±7	0.21/0.057/0.65
E/A	1.40±0.34	1.16±0.30	0.90±0.27	**0.012/<0.001/0.002**
Myocardial Mass, [g/m2]	123±41	194±71	210±69	**0.001/0.001**/1.00
SBP [mmHg]	119±8	128±14	129±15	**0.012/0.008**/0.454
DBP [mmHg]	78±8	84±8	85±9	0.024/0.033/0.526
Glucose [mg/dl]	85±18	93±11	94±11	0.14/0.06/0.45
Cholesterol [mg/dl]	204±60	219±39	206±46	0.20/1.00/0.54
LDL [mg/dl]	124±46	140±35	129±43	0.057/1.00/0.60
HDL [mg/dl]	55±11	56±15	56±14	1.00/1.00/1.00
BMI [kg/m2]	23.6±4.1	25.5±2.8	25.5±2.8	**0.002**/0.057/1.00

Significant differences are marked by bold letters.

### Differences in PCr/β-ATP Ratios between the Groups

**Figure 2:**
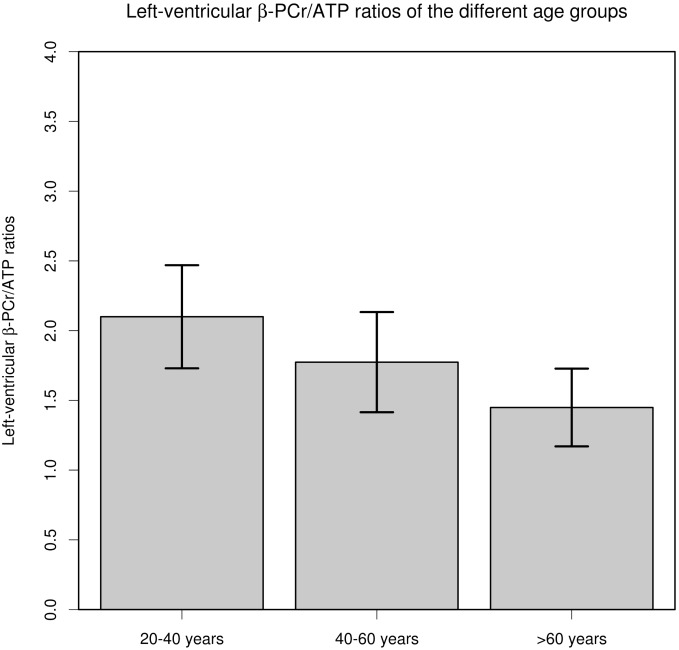
The bar plots shows the differences in means between the three age groups. The standard deviation are added as error bars. The differences in left-ventricular PCr/β-ATP ratios were significant (p<0.001) between all groups.

**Figure 3:**
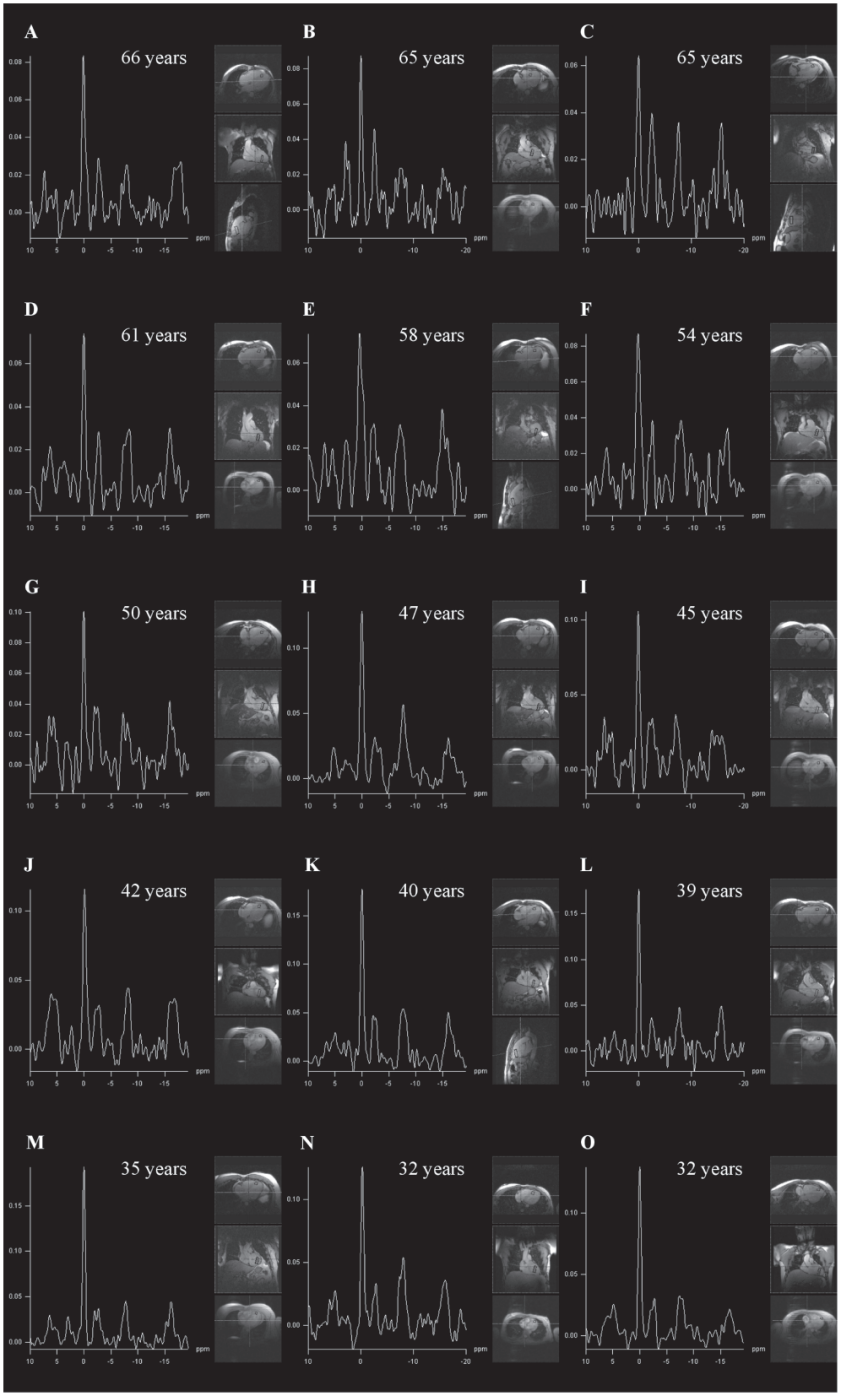
Representative spectra derived from the antero-septal myocardium of several volunteers are shown. Please note the tendency of decrease in phosphocreatine relative to ATP with increasing age.



As shown in Table 1 as well as in Figure 2, significant differences in PCr/β-ATP ratios were observed between all three groups. As suggested by Figure 3, the visual assessment of the left-ventricular MR spectra revealed a decline of the PCr peak with age in comparison to the β-ATP triplet. The Pearson’s correlation coefficient showed a significant correlation between age and PCr/β-ATP (r = −0.5; p<0.001), which was further evaluated by the linear regression analysis (Figure 1). The linear regression analysis (r^2^ = −0.25; p<0.001) revealed following relationship:

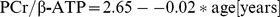



### Age and Cardiac Structure, Function and Metabolism

As demonstrated in Table 1, significant differences between all three groups were revealed in E/A, between group I and II as well as group I and III in MM and SBP, and only between group I and II in BMI. E/A (r = −0.502, p<0.001), MM (r = 0.304, p<0.001), glucose-levels (r = 0.157, p<0.05) and systolic blood pressure (SBP; r = 0.224, p<0.005) showed moderate to weak age-dependence. Moreover PCr/β-ATP ratios correlated significantly with MM (r = −0.317, p<0.001) and E/A (r = 0.213, p<0.02). The remaining measured laboratory and echocardiographic parameters did not show any significant correlation with left ventricular PCr/β-ATP ratios.

## Discussion

### Ageing and HEP Metabolism

The present study is a substantial extension of a previous publication of our study group [21]. In the present study, we investigated the impact of ageing on myocardial HEP metabolism in a nearly three-fold larger collective of healthy men. Our main finding was the linear relationship between age and left ventricular PCr/ATP ratios. Moreover, we divided our study population in three age groups and detected significant decreases in left ventricular PCr/ATP ratios between all groups. Two previous papers, however, reported on significant correlations between age and absolute concentrations of PCr and ATP, but not between age and PCr/ATP ratios in relatively small study populations, whereby the latter study group calculated absolute concentrations for PCr and γ-ATP, demonstrating moderate decreases in both with age, but not in the ratio [22]; [23]. The decrease in left-ventricular PCr concentrations are consistent with our data. However, the concomitant decrease in γ-ATP might not be comparable with our results. First, we did not determine absolute concentrations of the HEP in our collective. Second, we did not consider γ-ATP to reflect ATP, but formed ratios between PCr and β-ATP. The rationale for this procedure is the well known fact that the α- and the β-phosphates of adenosine-diphosphate (ADP) are located at −9.5 and −5.5 ppm, respectively [32]. The a-, β-, and γ-phosphates are commonly observed at −10.9, −21.2 and −5.7 ppm [33]. Furthermore, the a- and γ-position of ATP are not only contaminated by ADP, but also by NAD+/NADH at −10.5 ppm [34]; [35].

The question is whether the superposition of γ-ATP by β-ADP is a relevant factor. The determination of the ratio between ATP and ADP within cardiac myocytes is difficult. A previous publication in 1982 showed differences in myocardial ATP/ADP ratios, as measured within extracted samples by spectrophotometry and as calculated from 31P MRS data that vary from 5.7 to 534 [36]. Furthermore, the ATP and ADP concentrations within human erythrocytes are possibly relevant [34], whereby ADP within the blood is characterized by an extremely high visibility for 31P MRS [37]. Therefore, the ATP concentration, as measured within the myocardium by 31P MRS, is commonly corrected by the evaluation of 2,3-diphosphoglycerate (2,3-DPG) [11]; [18]. However, it appears to be complicated to correct the blood contamination of the ADP portion within the γ-ATP peak, whereby it remains unclear, whether these technical aspects may explain the differences between our results and previously published findings [22]; [23]. Fact is that our results are based on 196 healthy, asymptomatic male volunteers. Furthermore, a recent study showed also a significant correlation between left-ventricular PCr/ATP ratios and age in 49 healthy subjects, while myocardial energy metabolism did not correlate with left-ventricular function [24].

The metabolism of the heart is characterized by the remarkable stability of PCr and ATP, even if the respiration rate is increased due to exercise. This phenomenon is responsible for the fact that the state of fatigue is unknown for the healthy heart [38]. The basis for this metabolic homeostasis, also known as stability paradox, is a complex cellular regulation of mitochondrial respiration. In muscle tissue, creatine is rephosphorylated at the mitochondrial site to maintain a stable concentration of ATP at the myofibril site. In the beating myocardium, ATP is continuously splitted into ADP and inorganic phosphate by releasing [H+] ion in the cytoplasma, whereby the regeneration of ADP to ATP is catalyzed by the creatine kinase (CK) at the myofibril site and the elimination of inorganic phosphate and [H+] ions is managed by the mitochondria [1]; [39]; [40]. In both skeletal and cardiac muscle, the rephosphorylation of creatine to PCr, expressed by recovery rates or PCr concentrations in relation to ATP, characterize mitochondrial function [1]; [2]. Consequently, myocardial PCr/ATP ratios are an adequate method to quantify the capacity of rephosphorylation.

The main explanation for our results might be a loss of mitochondrial function and a decrease in oxidative phosphorylation with age. Accordingly, a previous experimental study has shown that the activity of CK decreases with age [41], which consecutively results in a decrease in cellular PCr levels [42]. Aside from impaired mitochondrial function due to enzyme failure, the myocardial lysosomes suffer from different age-related impairments and damages resulting in an insufficient autophagocytosis of defective mitochondria [27]; [28]. Furthermore, mitochondrial oxidant production increases with age resulting in oxidative modification of DNA, proteins and lipids within the mitochondria [26]. Those age-related cellular processes are supposed to cause functional failure and death of cardiac myocytes [27].

Consequently, our results fits excellently to the meanwhile well established hypothesis that myocardial mitochondrial function decreases with age. The linear regression analysis presented in our study suggests a decrease of 0.02 in the cardiac PCr/ATP ratio per year in middle-European people. Therefore, we could speculate that a hypothetical PCr/ATP of about zero might occur at an age of ∼130 years, which surprisingly well agrees with the longest life span of 122 years hitherto observed in the world [43]. Although the average life expectancy at birth has increased during the last centuries, the maximum life span has remained unchanged, which might be caused by accumulation of diverse, deleterious changes with time that increase the chance of disease and death [43]. Certainly, other publications of our study group detected a moderate impact of several cardiovascular risk factors on left-ventricular PCr/β-ATP ratios, which suggests that a healthy lifestyle could have some beneficial effect on cardiac metabolism [9]; [11]; [18]; [19].

### Ageing and Cardiac Function

As discussed above, ageing leads to mitochondrial and lysosomal impairment, which might be responsible for a reduced HEP metabolism [27]. Several experimental studies have shown that a de-arranged myocardial HEP metabolism results in alterations of cardiac structure and function [44] [45]; [46]. Nahrendorf et al. showed left ventricular hypertrophy and normal EF but reduced contraction velocity in CK knock-out mice (CK−/−) and concluded that hypertrophy is a mechanism of incomplete compensation for the absence CK [45]. Another recent, experimental study investigating left-ventricular function in several age groups of Fischer 344 x Brown Norway hybrid rats from six to 39 months detected an increase in left-ventricular mass-to-body weight ratio with age, a deterioration of systolic function, a decline in left-ventricular pressure and a gradual increase in fibrosis [47]. Therefore, the weak correlation of MM and E/A with PCr/ATP ratios in our study-group of asymptomatic, healthy volunteers might reflect a similar kind of compensation mechanism. Furthermore, several previous studies investigating cardiac function in healthy humans have detected decreases in E/A [21]; [29] probably due to accumulation of collagen within the myocardium [30] or reduced early diastolic left atrial pressure [48], in end-diastolic and end–systolic volumes as well as longitudinal left ventricular function [31]; [49]. Another aspect is that age-related thickening and stiffening of the large arteries cause increases in systolic blood pressure, while diastolic blood pressure generally declines after the sixth decade. Furthermore, the early diastolic filling rate declines 30–50% between the third and ninth decades, although the left-ventricular function remains relatively preserved [50]. The findings of these previous studies meet almost exactly our results of echocardiography and physical examination and confirm that ageing is one of the main causes for myocardial alterations leading to an increased risk of cardiac failure [27]; [28].

### Technical Aspects

Some limitations and technical aspects must be addressed. For 31P MRS, we used a surface coil providing a limited penetration range. Therefore, we could only measure the antero-septal area of the myocardium. According to previous cardiac 31P MRS studies, we corrected our spectroscopic data for T1 saturation effects caused by variations in repetition times due to ECG gating, for the NOE enhancement and for the blood contamination assuming that T1 relaxation times and NOE enhancement factors were not age-dependent [51]–[53]. For the evaluation of the cardiac HEP metabolism, we formed the ratio between PCr and β-ATP, which is an established physiological marker [54]. The ATP level was given by the β-ATP peaks, because the α-ATP and the γ-ATP signals are contaminated by other phosphates [34]. In addition, the left-ventricular PCr/β-ATP ratios, as determined in our collectives, were completely in the range of previously published, cardiac 31P MRS studies [55]. To our knowledge this is the first study reporting the use of two different scanner systems and 31P CSI protocols, which could possibly bias our results. Therefore, we performed a sub-analysis of 98 volunteers older than 48 [yrs] ( = median of study population) with a comparable age distribution and did not reveal significant differences or in PCr/ATP ratios. Furthermore, we correlated separately left-ventricular PCr/β-ATP ratios and age in both collectives and obtained similar results. Consequently, we also assumed that different 31P CSI protocols performed on 1.5 T systems from the same manufacturer are comparable, if the post-processing is identical, as already concluded by Bottomley et al. [56].

### Conclusion

PCr/ATP ratios in healthy males might be a function of age. which is probably due to mitochondrial ageing in heart muscle cells. A reduction of PCr/ATP is further associated with increasing myocardial mass and decrease in the E/A ratio in healthy volunteers, whereby the relationship between age and myocardial mass as well as E/A ratio corresponds to the literature, as mentioned above. However, this study is the first to demonstrate the relationship between myocardial energy metabolism and structural alterations in healthy humans. Therefore, we can speculate that the maximal lifespan is reached, when the left-ventricular PCr/ATP ratio is about zero. However, we also know from previous work that exercise capacity and cardiovascular risk factors can modify this ratio, which is an excellent indicator for the efficiency of energy metabolism within the myocardium. Accordingly, a previous study has shown that the mitochondrial oxidant production can be reduced by chronic exercise [57]. Since age remains still one of the most important cardiovascular risk factors, the measurement of the left-ventricular PCr/ATP ratio may serve as prognostic factor for the development of cardiovascular disease [58]. This aspect might become more important, when considering that cardiovascular diseases are on the rise and more than 70% of the population in the western developed countries have multiple cardiovascular risk factors [59].

## Methods

All included participants of this study served as controls in previous, already published studies that were approved by the local ethic committee.

### Study Population

In this study, 196 male volunteers (mean age: 47.6±11.2 years) without any history of cardiovascular or metabolic disorders were enrolled. 76 of these volunteers were already published by Schocke et al. [21]. The other subjects served as healthy controls in several studies of our group. Therefore, two collectives of healthy male volunteers were merged. All volunteers of both groups received cardiac 31P 2D CSI, transthoracal echocardiography and blood withdrawal for laboratory analysis. The blood samples were taken after a 8 h fasting period to evaluate lipid profiles and fasting-glucose levels. All laboratory tests were analyzed in the central laboratory of the Medical University of Innsbruck (MUI) according to international standards. Transthoracal echocardiography was performed with the help an Acuson ultrasound imaging system (Acuson, Sequoia C256, Siemens, Erlangen, Germany) equipped with a 3.5 MHz-transducer. Left ventricular (LV) volumes and ejection fraction (EF) were determined by using the modified Simpson method. Thickness of LV posterior wall and inter-ventricular septum were used to calculate left ventricular muscle mass (Penn formula). Left ventricular diastolic filling was evaluated by pulsed Doppler echocardiography by determination of the early (E) to atrial (A) peak ratio (E/A).

### 31P 2D CSI Protocols

**Figure 4:**
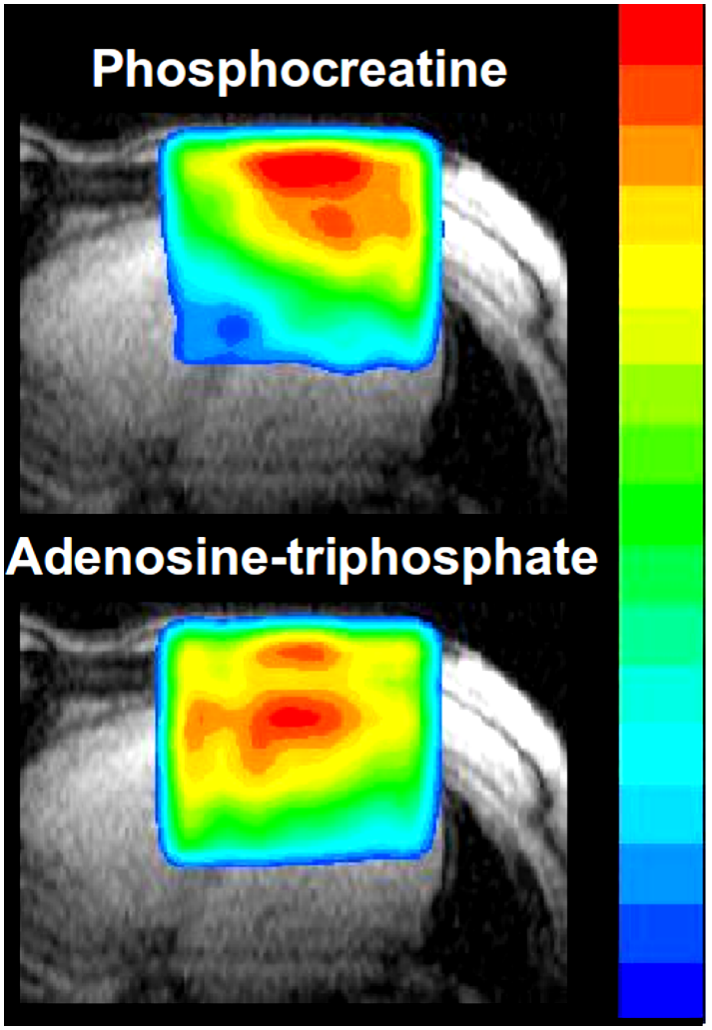
The representative metabolite images with color overlay are based on Fourier interpolation to a resolution of 64×64. Both the PCr and the β-ATP metabolite images show a clear separation between myocardium and skeletal muscle of the thorax. The myocardium is characterized by lower PCr concentrations and higher ATP concentrations compared to skeletal muscle, whereby the higher myocardial ATP concentration is partly caused by blood contamination.

The two collectives of healthy volunteers were measured on two different 1.5 Tesla whole-body MR scanners. The collective published in 2003 underwent 31P 2D CSI on a Magnetom Vision, the recent collective on the same Magnetom upgraded to Symphony standard (both Siemens Erlangen, Germany). For all volunteers, a circular, polarized, double resonator surface coil was used permitting the transmission and receipt of 1H resonance at 63.5 MHz and 31P resonance at 25.8 MHz. The transmitter coil had a diameter of 21 cm, the receiver coil a diameter of 14 cm. During the MR examination, all subjects were supine with the coil upon the thorax, providing a small gap between thorax muscles and myocardium (Figure 4).

**Figure 5:**
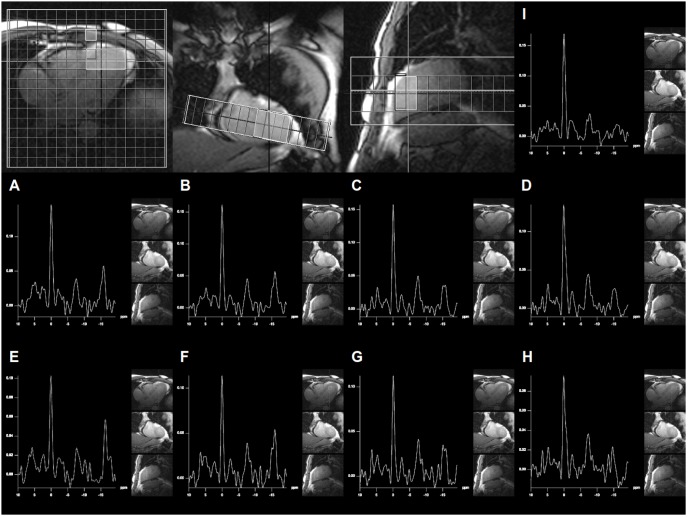
The montage of localizers and spectra are derived from a 62 year old, healthy man. The axial, coronal and sagittal images were acquired for localization purposes. The grid reflects the position of the MR spectroscopic slab, originally measured with 8×8 phase encoding steps, which were interpolated to a matrix of 16×16 in the k-space. The large, semi-transparent rectangles on the localizer images show the positions of spectra, which were averaged for the determination of left-ventricular PCr/β-ATP ratio (A–H). The small rectangle on the axial localizer image represents the position of a voxel from the thorax muscles, which is given for comparison. On each spectrum, pictograms of the localizer images are superimposed on the right margin.

**Figure 6:**
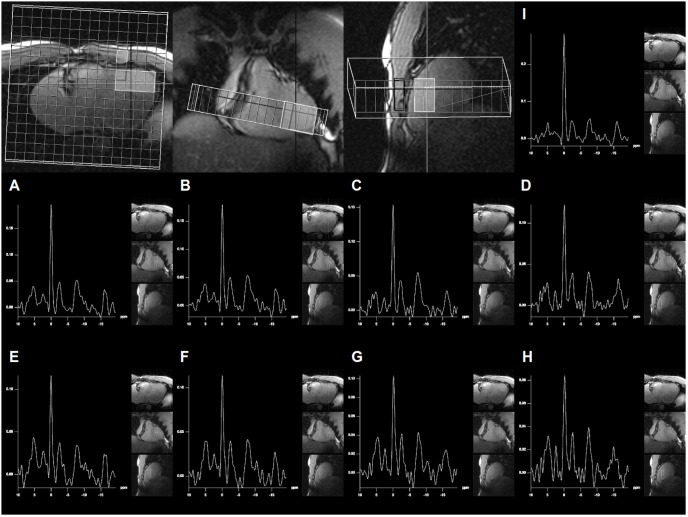
The montage of localizers and spectra are derived from a 32 year old, healthy man. The axial, coronal and sagittal images were acquired for localization purposes. The grid reflects the position of the MR spectroscopic slab, originally measured with 8×8 phase encoding steps, which were interpolated to a matrix of 16×16 in the k-space. The large, semi-transparent rectangles on the localizer images show the positions of spectra, which were averaged for the determination of left-ventricular PCr/β-ATP ratio (A–H). The small rectangle on the axial localizer image represents the position of a voxel from the thorax muscles, which is given for comparison. On each spectrum, pictograms of the localizer images are superimposed on the right margin.

The sequence parameters for the 31P 2D CSI measurements of both collectives were previously published. For cardiac 31P MRS, we used a cardiac gated, transversal 31P 2D CSI sequence with nucleus Overhauser enhancement (NOE) and an excitation delay of approximately 100 to 120 ms to the R-wave in both collectives. The repetition time depended on the R-R interval of the volunteers during the MRS examination. The sequence parameter of the 31P 2D CSI sequence on the Magnetom Vision comprised a field of view of 320 mm, 8×8 phase encoding steps, an echotime of 3 ms, a flip angle of 90°, a slab thickness of 40 mm and a signal collection over 16 acquisitions. Before inverse Fourier transformation in the two k-space directions raw data were interpolated to a matrix of 32×32 by using zero filling. The 31P 2D CSI sequence used on the Magnetom Symphony had a field of view of 200 mm, 8×8 phase encoding steps, an echotime of 2.3 ms, a flip angle of 90°, a slab thickness of 40 mm and permitted acquisition weighted measurements with a signal collection over 64 acquisitions. The raw data were interpolated to a matrix of 16×16 (Figure 5 and 6).

After Fourier transformation, frequency shift, phase and baseline correction were applied in the frequency-domain. Post processing was performed on a standard LEONARDO-console using a standard software-package (Siemens Erlangen, Germany). After optimization of phase correction, fitting under the curve was done for the peaks of PCr, β-ATP and 2,3-diphosphoglycerate (2,3-DPG). Results of integrals under the spectra were further corrected for blood contamination, T1- saturation effects and NOE as previously published [11]; [21]. The PCr/β-ATP ratios were calculated for 8 voxels of the left ventricle, summed and then averaged, in order to determine myocardial HEP metabolism.

### Statistical Analysis

Analyses were performed by employing the open-source statistical software R [60]. The significance level was set at <0.05. Normal distribution was tested by the Kolmogorov-Smirnov test. SBP, DBP and glucose levels were not normally distributed. Normally distributed data were are presented as mean ± standard deviation (SD) and tested with the help of parametric tests, whereas not normally distributed data were expressed as median and range and further evaluated with the help of non-parametric tests. the univariate ANOVA with Bonferroni post-hoc testing was used to determine differences in the normally distributed parameters between the three groups. In case of not normally distributed parameters, overall significant effects were assessed with the Kruskal-Wallis test followed by posthoc Mann-Whitney-U test. For this purpose the significance level was corrected to <0.017. Correlation analyses were performed with the help of the Pearson’s (if normally distributed) or the Spearman’s (if not normally distributed) correlation coefficients. A correlation coefficient *r* of 0.35–0.49 was interpreted empirically as low, 0.5–0.79 as moderate and 0.8 or greater as high. Moreover, the linear regression analysis was employed for selected parameters.
